# Tea at the Hall: Some Recollections of a Far-Distant Final

**Published:** 1912-09-07

**Authors:** 


### TEA AT THE HALL.

### Some Recollections of a Far-distant Final.

The old Examination Hall on the Victoria Embankment no longer attracts the shivering medical student. It has been given over to the Electric Engineers and the L.C.C., and the Conjoint Board houses in Bloomsbury. Yet which of us who have visited its rooms for at least the stipulated three times can pass it without feeling something of the sentimental respect that the student attaches to his Alma Mater! If there is one building in England that can lay claim to being the universal Alma Mater of the profession, it is the Embankment Hall. There was always an element of uncertainty; perhaps for that very reason the pleasure was the greater. But at least there was always good-will, gentlemanliness, and honesty. Legends there are that speak of antagonistic conditions, but they are legends merely. Who credits the story that a candidate once got " three years " for having hit an idiotic and bullying examiner in the eye? Or the triumphant tag to it that the Board induced the said examiner to apologise to the candidate and then resign?

### While-away Stories.

What stories whiled away the waiting hours in the outside room of the Hall below in the afternoons when the final was on! The writer recollects, in his own case, how when moodily striding up and down the room in sheer agony of mind because of a possible " howler " in answer to a catch question, he was accosted by a fellow-student, up to that moment unknown to him, who commenced by detailing the cases " up " from his own hospital. " There's a Johnny with a syphilitic liver," he remarked. " Old S—— says you'll all go for malignant disease. And look out for the eye case. There's only two, and one is ours. Its a keratitis with commencing cataract." Usually the tips proved unreliable; now and then good advice was given—by accident very likely. How many of us wasted time and energy in looking up " certainties " for the paper which were never answered! Yet it was remarkable that a few really forecasted surely, helped probably by subconscious hints from the lecturers.

All these things probably happen to-day in Bloomsbury, and will continue to happen so long as examinations last. Candidates will interchange comments on the merits of the respective examiners. " So and so knows no anatomy—he always reads up a few catches and springs them on you." " Old M—— is sure to ask you the function of the popliteus. He always does." And in the dusk, sitting on the window-sill that commanded a view of the Thames and the Needle, how many of us revealed secrets of personal interest anent our vivas, in some cases drawing very much on our imaginations. " They wanted me to do an excision of the wrist. I said 'All right,' but I couldn't do it within a quarter of an hour. So they gave me a Stokes-Gritti, which I finished in two minutes, my boy." Some day some one will write the history of the Embankment Hall and put on record all these stories. Poor ones many, no doubt, but, like Oliver Wendell Holmes' trees, all interesting to those who interest themselves in such things. Some, even, with glimpses of rare humour in them. Is there not a tale of the examiner who was always asking candidates to tell him the specific gravity of spermatocele fluid until his colleague got annoyed and asked, in his turn, a flabbergasted candidate, " What, pray, is the specific gravity of the fluid in the semi-circular canals? " " I don't know," said the truthful candidate. " Neither do I," commented the colleague, " and I don't think it matters in the least "—a comment which it is to be hoped his meticulous-minded " marker " took to heart.

### The Candidates' Tea.

The event of the day at the Hall was afternoon tea. The janitor and his small myrmidon brought this into the room; plates of thin bread and butter, sponge and currant cake, and a monstrous urn with many cups, a big sugar basin, and one teaspoon to every three candidates. It was a study in psychology to watch how the men took their tea. Some pleasantly, as if it was an ordinary occasion and they had no qualms whatsoever; others jerkily, the spilt fluid and crumbled cake testifying to their mental agitation; others, again, stolidly, with a Socrates-like resignation that nothing could shake. Above the murmur of conversation—limited chiefly to comments on the vivas and forecasts of results—came the monotonous calls of the janitor, " Numbers 265, 266, 267, and 268," or other fours in similar sequence. The candidates called upon would march upstairs and disappear, to return from the back an hour or so later, when they would give the smaller crowd of colleagues still waiting in misery the benefit of their experience. The tea was always appreciated—by those who drank it, for some preferred a stroll on the Embankment or a visit to a neighbouring Lyons, or even to the adjacent Coal Hole.

And then the evening waits. The speculation about whose car it was that waited outside: the dreary wait for the final batch of candidates to come down! There was excitement in the air. Only a Dickens, fresh from his description of Carton's ride to the scaffold, could do justice to that wait. The signing of the diplomas and the march in to meet the Examining Board constituted almost an epic—an epic in which comedy and tragedy mingled to an extent that defied the rules of the game as understood by Milton and Camoes.

The passing of the Embankment Hall is like the death of an old friend. It is true the building held no such friendly memories as the medical school or the hospital itself, but it was consecrated by other recollections than those which clustered to college or ward. Its lustre and glamour will doubtless pass to the new Hall in Bloomsbury, which is the goal for the coming generations of medical men—possibly even the home of the one portal examination that most of us dream of.

